# Ethical HIV research with transgender and non‐binary communities in the United States

**DOI:** 10.1002/jia2.25971

**Published:** 2022-10-12

**Authors:** Augustus Klein, Sarit A. Golub

**Affiliations:** ^1^ Hunter Alliance for Research and Translation Hunter College New York City New York USA; ^2^ Department of Psychology Hunter College New York City New York USA; ^3^ Department of Basic and Applied Social Psychology The Graduate Center of the City University of New York New York City New York USA

**Keywords:** HIV prevention and treatment, community‐engaged research, transgender, non‐binary, stigma, ethical considerations

## Abstract

**Introduction:**

Because transgender individuals experience disproportionately high rates of HIV infection, this population is an increasing focus of epidemiological and implementation science research to combat the epidemic. However, study participants, providers and other advocates have become increasingly concerned about research practices that may alienate, objectify, exploit or even re‐traumatize the communities they are designed to benefit. This commentary explores the common pitfalls of HIV research with transgender communities and provides a potential framework for ethical, community‐engaged research practice.

**Discussion:**

We review some of the critical challenges to HIV research with transgender and non‐binary communities that limit the potential for such studies to improve practice. For example, scales that measure stigma perceptions/experiences often include activating language, while the consistent focus in research on risk and trauma can often feel judgemental and redundant. Because of limited employment opportunities, some participants may feel undue influence by research stipends; others may perceive their participation as fuelling the larger research economy without providing research jobs to community members. Questions remain regarding optimal strategies for authentic research partnership beyond community advisory boards or focus groups. Transgender and non‐binary researchers are under‐represented and may be tokenized. Many demonstration projects provide much‐needed services that disappear when the research funding is over, and community‐based dissemination efforts are often perceived as “too little, too late” to effect change.

**Conclusions:**

Based on this review and input from study participants across the United States, we detail six recommendations for ethical HIV research with transgender and non‐binary communities, including (1) equitable budgeting with community‐based programme partners; (2) representation in the development of both research agenda and methods; (3) integration of research activities into the ongoing work of any clinical or service site, so that individuals’ needs as “clients” can continue to be prioritized over their role as “participants;” (4) mindfully considered compensation that values the contributions of community members, but avoids undue influence; (5) transparent, community‐focused and timely communication at every stage of the study, including research purpose, data usage, preliminary findings and full‐scale results; and (6) planning for sustainability of any programme or services beyond the life of the research project.

## 1 INTRODUCTION

Over the past two decades, transgender individuals, particularly African American/Black and Latinx/Hispanic transgender women, have received increasing attention in HIV research [1, 2, 3, 4, 5, 6, 7, 8]. This research has documented how multiple intersecting structural factors, including racism, sexism, transphobia, homophobia and other systems of oppression, contribute to the disproportionately high rates of HIV infection among this population [9, 10, 11, 12, 13, 14]. However, the proliferation of transgender HIV research has brought significant scientific and ethical concerns about research practices that may erase, alienate, objectify, exploit or re‐traumatize the communities they are designed to benefit [7, 13, 15–18]. A growing number of voices within the transgender and non‐binary communities (TGNBCs) have begun to openly challenge the scientific rigour, public health benefit, ethical principles and research practice within trans‐focused HIV science by advocating for systemic change that prioritizes, promotes and sustains meaningful trans leadership and engagement at all levels of the research process [15, 19–24].

As part of this challenge, it is essential to acknowledge the diversity of populations outside a traditional cisgender focus. While the focus has been on transfeminine people in HIV research, transmasculine individuals have historically been marginalized due to assumptions about their sexual partners and activities, low estimates of HIV prevalence compared to transgender women and a lack of adequate and standardized HIV surveillance data collection about gender identity. Non‐binary and gender diverse individuals are almost entirely absent from research representation due to binary assumptions about gender identity, a lack of expansive measures of gender identity in existing research and the conflation of sexual and gender identity. This commentary explores the persistent problems and critical challenges of HIV research with TGNBCs and provides a potential framework for ethical, community‐engaged research practice.

## 2 DISCUSSION

Table 1 presents six methodological and ethical concerns in HIV research with TGNBCs and identifies the critical challenges our field must embrace to make equity‐focused progress. This analysis emerges from our combined 30 years of experience working in community‐based healthcare, social services and advocacy within HIV and our more recent experience conducting community‐driven implementation science research. Most recently (September 2020–August 2021), we facilitated a convening of five community health centres across the United States serving LGBTQ+ communities. We engaged in a multi‐stage collaborative process that included over 350 diverse stakeholders (over 50% of whom were patients) and culminated in a 4‐day virtual convening in which 49 representatives from all five health centres (41% patients; 34% transgender/non‐binary; and 49% People of Colour) came together to discuss the past and future of equity‐focused, community‐engaged research. We acknowledge this history as the lens through which we understand and articulate the concerns and challenges below. We invite others with distinct and complementary perspectives to engage, critique and add to the conversation.

**Table 1:** Critical challenges to addressing methodological and ethical concerns in HIV research with transgender and non‐binary individuals

Methodological and ethical concerns	Critical challenges
Overemphasis on descriptive documentation of risk, pathology and “vulnerabilities” among transgender and non‐binary communities.	Committing to integrated, multi‐level approaches that emphasize contextual drivers of individual‐level behaviour and focus on identifying targets for disruption of intersectional structural oppression.
Continuous recapitulation of known facilitators and barriers to HIV prevention and treatment.	Empowering researchers not to re‐establish barriers and facilitators but instead identify and test strategies for addressing them.
Studies that examine stigma and trauma may include activating, re‐traumatizing language and/or may be measured in ways that feel judgemental, out of touch and demoralizing.	Ensuring that research methods are trauma‐informed and actively de‐stigmatizing.
Lack of reflection about power dynamics and potential undue influence inherent in research compensation.	Developing compensation strategies that recognize and value contributions without being exploitative.
Under‐representation or tokenization of transgender and non‐binary individuals in all stages of the research process.	Developing structures and accountability for genuine research partnership that promotes and sustains leadership of transgender and non‐binary individuals.
Lack of durable impact of HIV research on transgender and non‐binary communities.	Ensuring sustained access to needed programmes or services and multi‐level dissemination that focuses on maximizing the impact of research findings.

### 2.1 Methodological and ethical concerns

The first concern is the extent to which trans‐HIV research has emphasized descriptive documentation of risk, pathology and “vulnerabilities” among TGNBCs associated with potential HIV exposure, including sex work, lack of employment opportunities, housing instability, incarceration, violence, substance use and depression [7, 11, 13, 17, 25, 26]. On the one hand, this focus has enabled a greater understanding of several acute challenges faced by TGNBCs, and some findings have been used as essential advocacy tools. However, research focused merely on *describing* health disparities has the potential to further stigmatize TGNBCs by reducing them to their marginalized identities and the contextual factors they must navigate to survive. Moreover, much of this research does not adequately analyse systemic drivers of HIV exposure; for example, in a recent scoping review of articles measuring anti‐trans stigma in the United States [27], only 35/126 (28%) measured stigma at the structural level and only four (3%) provided a specific definition of structural anti‐trans stigma. One critical challenge in this area is committing to integrated, multi‐level approaches that emphasize contextual drivers of individual‐level behaviour and focus on *identifying targets* for disrupting intersectional structural oppression.

Relatedly, the second concern is the continuous recapitulation of known facilitators and barriers to HIV prevention and treatment for TGNBCs. Almost all publications in this area document the same barriers and facilitators, including healthcare access, financial need, social support and intersecting multi‐level stigma and discrimination [28, 29, 30, 31, 32, 33, 34, 35]. However, this line of inquiry has failed to fully identify specific mechanisms and manifestations that could serve as intervention targets for increasing engagement and equity. For example, little attention has been given to examining the processes and mechanisms *within HIV treatment and prevention programmes* that perpetuate structural oppression and contribute to a lack of access and uptake of services, such as the continued use of HIV risk assessment tools, outreach materials or programme delivery models that “lump” TGNBC together with gay men [36, 37]. One critical challenge is empowering researchers to accept existing research on healthcare access among TGNBCs as a framework for intervention development without re‐establishing identical or similar barriers and facilitators in each research context. We must prioritize research that identifies and tests strategies for addressing known barriers and facilitators to care, accelerating research designed to solve the problems we already know exist.

The third concern is the extent to which some research strategies may recreate or activate the traumatic or stigmatizing experiences they are designed to understand. Stigma and minority stress scales [38, 39, 40, 41] often include items that can be experienced as stigmatizing in and of themselves (e.g. “Whenever I think of being transgender, I feel depressed;” “Most transgender people end up lonely and isolated;” “Being transgender is disgusting to me”) [40] or ask participants to recount and relive stigmatizing experiences (e.g. history of physical and sexual violence, family rejection, incidents of discrimination and harassment) to document their association with negative affect or health behaviour [5]. While some studies complement these measures with measures of self‐esteem, community‐connectedness or other “protective factors,” [42, 43] there is limited research analysing how the remarkable strength and resilience exhibited by TGNBCs in response to systemic oppression can be translated into specific supportive interventions and programmes. To move towards a strengths‐based health equity framework, it is incumbent on researchers to consider the reasons why we are asking certain research questions (especially if we already know the answers) and whether the utility of including certain measures outweighs the potential harm to study participants. One critical challenge for the future is to ensure that research methods are trauma‐informed and actively de‐stigmatizing by (1) focusing on resiliency, strength and joy; (2) ensuring that study language is person‐centred and non‐stigmatizing; and (3) building in active support mechanisms for TGNBCs participating in studies dealing with stigma and trauma.

The fourth concern relates to the need to be reflective and deliberate about research compensation in the context of a complicated risk/benefit calculus for many TGNBC research participants. From the researcher's perspective, participation is compensated for the time and travel involved in “data collection,” not the participant's information. However, given the extent to which financial need has been identified as a particular vulnerability of TGNBCs, our community partners encourage researchers to be particularly vigilant about the potentially predatory nature of collecting highly sensitive and often traumatic information in exchange for monetary compensation. Our community partners recommend two strategies to offset concerns about power dynamics and undue influence. First, participants want to feel motivated to participate in research even if they are not being financially compensated. To the extent that TGNBCs think that the research mission and findings have the potential to benefit their community, financial compensation for their time makes them feel valued rather than exploited. Second, participants want to be offered other incentives in addition to monetary compensation, such as access to resources or services, connection to other participants as a form of social support and opportunities to shape research progress. One critical challenge for the future is the development of creative compensation strategies that recognize and value participant contributions without undue influence.

The fifth concern is the under‐representation or tokenization of TGNBCs at all levels of the research process [7, 15, 20–22]. In the current landscape of HIV research, cisgender researchers receive much of the funding for transgender research [15]. Despite a growing number of TGNB researchers trained in academia or community‐based settings, there are few opportunities for genuine professional development, training and mentorship [15, 21]. When TGNBCs are represented in research, they are often relegated to community advisor or “consultant” roles and asked to speak for or connect researchers to “the community” only once funding has been received, and the study design is complete [15, 20–22]. This practice undervalues or erases the substantive training and expertise of TGNBCs, reducing their importance to their willingness to perform their transgender identity. One critical challenge is for researchers and funders to develop accountability structures for genuine research partnerships that promote and sustain TGNBC leadership and involvement at all stages of the research process.

The sixth concern is the lack of durable impact of HIV research on communities. This concern manifests along two dimensions. First, many HIV intervention or demonstration projects provide much‐needed services (e.g. pre‐exposure prophylaxis) to TGNBCs that disappear when research funding ends [19]. From the research perspective, such studies are conceptualized as a means to an end, intended to generate findings that can be used to enhance care at some point in the future. But this perspective neglects the short‐term reality that research participants will lose access to vital resources and services. Second, even community‐based research projects are often not “nimble” enough to respond to the immediate or shifting needs of TGNBCs. Due to the urgent need for competent healthcare, TGNBCs cannot wait years to learn whether a particular intervention or treatment is effective. Many community‐based dissemination efforts are perceived as “too little, too late” to effect change. One critical challenge is for all research projects to include (1) a dissemination plan that can provide ongoing reports of study findings in an accelerated time frame designed to maximize impact; (2) an adaptability plan that can pivot in response to new developments or unexpected study findings; and (3) a sustainability plan, which ensures that efficacious services offered as part of a research study can be sustained after the research is completed.

## 3 CONCLUSIONS

To address the methodological and ethical concerns discussed in this commentary, HIV research must create mechanisms to centre the values, needs and priorities of TGNBCs at all stages of the research process. Without this commitment, HIV research will continue to perpetuate the inequities it has been funded to address. The following recommendations below represent six “take‐home” messages that we believe are most important to consider. While these recommendations do not represent a one‐to‐one relationship to the specific concerns detailed above, they are provided as an overarching framework to begin addressing these critical challenges.

1. **Ensure equitable budgeting with community‐based programme partners**. Community‐based organizations (CBOs) serving TGNBCs play an integral role in HIV research. However, CBOs are inherently disadvantaged in legal and financial power when receiving research requests from outside researchers, particularly at large academic institutions. Many institutions which fund HIV research perpetuate this power dynamic by not requiring researchers to actively consider the programmatic and financial investment necessary for organizations to successfully support and engage in research, such as staffing, prioritization of care provision versus research responsibilities, service reimbursement structures and the technological infrastructure needed for data collection or abstraction. CBOs which provide services to TGNBCs most impacted by HIV are often underfunded, understaffed and politically vulnerable. HIV research that centres health equity must intervene at the structural and programmatic level by designing equitable compensation structures that are based on (1) the amount of research funding received; (2) the organization's mission, infrastructure and services provided; and (3) the research responsibilities and expectations requested.
2. **Increase representation of TGNBCs in developing both research agenda and methods**. Central to meeting all critical challenges outlined in this commentary is a renewed commitment from HIV researchers to create and foster true research partnerships and for funders to require and incentivize formal research engagement plans that concretely outline how TGNBCs will participate at all stages of the research process.
3. **Integration of research activities into the ongoing work of CBOs, focusing on prioritizing individuals’ roles as “clients” over their requirements as “participants.”** The co‐location of research within CBOs can answer research questions that are most pertinent to TGNBCs and allow organizations to put research findings into action in a timely and sustainable manner, providing an opportunity to build a rigorous evidence base for effective practice‐based strategies/interventions that have a lasting impact on the lives of TGNBCs. However, in this context, researchers must ensure that research needs do not eclipse or threaten patient care. Funders and researchers must value and prioritize research that actively engages CBOs as research leaders and acknowledges the importance of the care they provide.
4. **Mindfully considered compensation that values the contributions of community members but avoids undue influence**. As discussed above, compensation strategies must align with the tasks participants are asked to complete and the types of information being collected. Compensating participants does not mean we can continually ask intimate, stigmatizing questions. There is a need for greater transparency around the reasons for asking such questions and how this information will be used to benefit study participants and the community. It is also incumbent on researchers to consider providing opportunities for research participants beyond financial incentives. For example, participation in research can be an opportunity for forging connections among individuals with shared experiences and interests. Researchers should consider innovative ways to provide much‐needed social support and community‐building opportunities.
5. **Transparent, community‐focused and timely communication at every stage of the research process**. Conceptualizing dissemination as a process that begins at the inception of a research project and continues throughout implementation is a key to meeting the critical challenges outlined in this commentary that demand *timely and useful* research for TGNBCs. Dissemination activities and protocols should (1) build trust around and enhance the applicability of findings; (2) include standardized “report‐back” protocols for timely and ongoing dissemination of study information and findings; (3) facilitate real‐time feedback, questions and concerns from study participants and programme staff; and (4) ensure study information and findings reach beyond academic and scientific communities.
6. **Planning for the sustainability of any programme or service beyond the life of the research project**. To ensure that HIV research with TGNBCs does not perpetuate the health disparities it has documented, it is vital for all research projects that provide access to services or treatment to develop and execute a plan for sustainability of services once the project has ended. This work requires creative, thoughtful pre‐planning in collaboration with CBOs and TGNBCs. Funders could require sustainability plans as a core component of ethical practice and sustainability in the event of efficacious findings.

The above recommendations are based on our ongoing work and input from TGNBCs, including study participants, researchers and healthcare providers across the United States. We encourage the reader to reflect on how the above recommendations may be implemented to enhance current and future research. The ideas presented in this paper are designed to be a call to action for improving ethical, community‐based research practice with TGNBCs that is best equipped to enhance health equity in HIV prevention and treatment. The overarching challenge for our field is incentivizing action‐oriented research that centres health equity by developing and evaluating real‐world strategies that mitigate against inequity in HIV prevention and care access, utilization and efficacy among TGNBCs.

## COMPETING INTERESTS

The authors declare no competing interests.

## AUTHORS’ CONTRIBUTIONS

Both AK and SAG developed the concept for this paper and contributed to the writing, revision and table development.

## References

[1] Herbst JH , Jacobs ED , Finlayson TJ , McKleroy VS , Neumann MS , Crepaz N , et al. Estimating HIV prevalence and risk behaviors of transgender persons in the United States: a systematic review. AIDS Behav. 2008;12(1):1–7. 17694429 10.1007/s10461-007-9299-3

[2] Baral SD , Poteat T , Strömdahl S , Wirtz AL , Guadamuz TE , Beyrer C. Worldwide burden of HIV in transgender women: a systematic review and meta‐analysis. Lancet Infect Dis. 2013;13(3):214–22. 23260128 10.1016/S1473-3099(12)70315-8

[3] Poteat T , Reisner SL , Radix A. HIV epidemics among transgender women. Curr Opin HIV AIDS. 2014;9(2):168. 24322537 10.1097/COH.0000000000000030 PMC5947322

[4] Clark H , Babu AS , Wiewel EW , Opoku J , Crepaz N. Diagnosed HIV infection in transgender adults and adolescents: results from the National HIV Surveillance System, 2009–2014. AIDS Behav. 2017;21(9):2774–83. 28035497 10.1007/s10461-016-1656-7 PMC5491368

[5] Becasen JS , Denard CL , Mullins MM , Higa DH , Sipe TA. Estimating the prevalence of HIV and sexual behaviors among the US transgender population: a systematic review and meta‐analysis, 2006–2017. Am J Public Health. 2019;109(1):e1–8. 10.2105/AJPH.2018.304727 PMC6301428 30496000

[6] Reisner SL , Murchison GR. A global research synthesis of HIV and STI biobehavioural risks in female‐to‐male transgender adults. Glob Public Health. 2016;11(7–8):866–87. 26785800 10.1080/17441692.2015.1134613 PMC4993101

[7] Del Río‐González AM , Lameiras‐Fernández M , Modrakovic D , Aguayo‐Romero R , Glickman C , Bowleg L , et al. Global scoping review of HIV prevention research with transgender people: transcending from trans‐subsumed to trans‐centered research. J Int AIDS Soc. 2021;24(9):e25786. 34473421 10.1002/jia2.25786 PMC8412127

[8] Stephenson R , Riley E , Rogers E , Suarez N , Metheny N , Senda J , et al. The sexual health of transgender men: a scoping review. J Sex Res. 2017;54(4–5):424–45. 28140660 10.1080/00224499.2016.1271863

[9] Brennan J , Kuhns LM , Johnson AK , Belzer M , Wilson EC , Garofalo R , et al. Syndemic theory and HIV‐related risk among young transgender women: the role of multiple, co‐occurring health problems and social marginalization. Am J Public Health. 2012;102(9):1751–7. 22873480 10.2105/AJPH.2011.300433 PMC3416048

[10] Lemons A , Beer L , Finlayson T , McCree DH , Lentine D , Shouse RL , et al.; Medical Monitoring Project . Characteristics of HIV‐positive transgender men receiving medical care: United States, 2009–2014. Am J Public Health. 2018;108(1):128–30. 29161069 10.2105/AJPH.2017.304153 PMC5718928

[11] Neumann MS , Finlayson TJ , Pitts NL , Keatley J. Comprehensive HIV prevention for transgender persons. Am J Public Health. 2017;107(2):207–12. 27997228 10.2105/AJPH.2016.303509 PMC5227924

[12] Poteat T , Malik M , Scheim A , Elliott A. HIV prevention among transgender populations: knowledge gaps and evidence for action. Curr HIV/AIDS Rep. 2017;14(4):141–52. 28752285 10.1007/s11904-017-0360-1 PMC5896563

[13] Sevelius JM , Reznick OG , Hart SL , Schwarcz S. Informing interventions: the importance of contextual factors in the prediction of sexual risk behaviors among transgender women. AIDS Educ Prev. 2009;21(2):113–27. 19397434 10.1521/aeap.2009.21.2.113 PMC4535696

[14] Operario D , Nemoto T. HIV in transgender communities: syndemic dynamics and a need for multicomponent interventions. J Acquir Immune Defic Syndr. 2010;55(2):S91. 21406995 10.1097/QAI.0b013e3181fbc9ec PMC3075534

[15] Scheim AI , Appenroth MN , Beckham SW , Goldstein Z , Grinspan MC , Keatley JG , et al. Transgender HIV research: nothing about us without us. Lancet HIV. 2019;6(9):e566–7. 31439535 10.1016/S2352-3018(19)30269-3 PMC7211382

[16] Perez‐Brumer AG , Oldenburg CE , Reisner SL , Clark JL , Parker RG. Towards ‘reflexive epidemiology’: conflation of cisgender male and transgender women sex workers and implications for global understandings of HIV prevalence. Glob Public Health. 2016;11(7–8):849–65. 27173599 10.1080/17441692.2016.1181193 PMC4931972

[17] Poteat T , Wirtz AL , Radix A , Borquez A , Silva‐Santisteban A , Deutsch MB , et al. HIV risk and preventive interventions in transgender women sex workers. Lancet North Am Ed. 2015;385(9964):274–86. 10.1016/S0140-6736(14)60833-3 PMC4320978 25059941

[18] Radix AE , Harris AB , Goldstein ZG. How can we improve uptake of oral HIV pre‐exposure prophylaxis for transgender individuals? Expert Rev Anti Infect Ther. 2020;18(9):835–8. 32316773 10.1080/14787210.2020.1759418

[19] Reisner SL , Radix A , Deutsch MB. Integrated and gender‐affirming transgender clinical care and research. J Acquir Immune Defic Syndr. 2016;72(3):S235. 27429189 10.1097/QAI.0000000000001088 PMC4969060

[20] Adams N , Pearce R , Veale J , Radix A , Castro D , Sarkar A , et al. Guidance and ethical considerations for undertaking transgender health research and institutional review boards adjudicating this research. Transgend Health. 2017;2(1):165–75. 29098202 10.1089/trgh.2017.0012 PMC5665092

[21] Rosenberg S , Tilley PM. ‘A point of reference’: the insider/outsider research staircase and transgender people's experiences of participating in trans‐led research. Qual Res. 2021;21(6):923–38.

[22] Vincent BW. Studying trans: recommendations for ethical recruitment and collaboration with transgender participants in academic research. Psychol Sex. 2018;9(2):102–16.

[23] Seekaew P , Janumnuaysook R , Lujintanon S , Pongtriang P , Nonnoi S , Hongchookait P , et al. Perspectives of transgender women toward study recruitment and retention: thematic analysis from Thailand. Sex Res Soc Policy. 2022;19(2):638–46.

[24] Veale JF , Deutsch MB , Devor AH , Kuper LE , Motmans J , Radix AE , et al. Setting a research agenda in trans health: an expert assessment of priorities and issues by trans and nonbinary researchers. Int J Transgend Health. 2022;19:1–7. 10.1080/26895269.2022.2044425 PMC9621229 36324879

[25] Fletcher JB , Kisler KA , Reback CJ. Housing status and HIV risk behaviors among transgender women in Los Angeles. Arch Sex Behav. 2014;43(8):1651–61. 25190499 10.1007/s10508-014-0368-1 PMC4214608

[26] Reback CJ , Fletcher JB. HIV prevalence, substance use, and sexual risk behaviors among transgender women recruited through outreach. AIDS Behav. 2014;18(7):1359–67. 24287786 10.1007/s10461-013-0657-z PMC4209944

[27] King WM , Hughto JM , Operario D. Transgender stigma: a critical scoping review of definitions, domains, and measures used in empirical research. Soc Sci Med. 2020;250:112867. 32163820 10.1016/j.socscimed.2020.112867 PMC7442603

[28] Fontanari AM , Zanella GI , Feijó M , Churchill S , Lobato MI , Costa AB. HIV‐related care for transgender people: a systematic review of studies from around the world. Soc Sci Med. 2019;230:280–94. 31035207 10.1016/j.socscimed.2019.03.016

[29] Pacífico de Carvalho N , Mendicino CC , Cândido RC , Alecrim DJ , Menezes de Pádua CA . HIV pre‐exposure prophylaxis (PrEP) awareness and acceptability among trans women: a review. AIDS Care. 2019;31(10):1234–40. 31043069 10.1080/09540121.2019.1612014

[30] Andrasik MP , Yoon R , Mooney J , Broder G , Bolton M , Votto T , et al. Exploring barriers and facilitators to participation of male‐to‐female transgender persons in preventive HIV vaccine clinical trials. Prev Sci. 2014;15(3):268–76. 23446435 10.1007/s11121-013-0371-0 PMC3760994

[31] Lacombe‐Duncan A , Kia H , Logie CH , Todd KP , Persad Y , Leblanc G , et al. A qualitative exploration of barriers to HIV prevention, treatment, and support: perspectives of transgender women and service providers. Health Soc Care Community. 2021;29(5):e33–46. 33237600 10.1111/hsc.13234

[32] Auerbach JD , Moran L , Watson C , Weber S , Keatley J , Sevelius J. We are all women: barriers and facilitators to inclusion of transgender women in HIV treatment and support services designed for cisgender women. AIDS Patient Care STDs. 2020;34(9):392–8. 32813571 10.1089/apc.2020.0056 PMC7480717

[33] Maiorana A , Sevelius J , Keatley J , Rebchook G. “She is like a sister to me.” Gender‐affirming services and relationships are key to the implementation of HIV care engagement interventions with transgender women of color. AIDS Behav. 2021;25(1):72–83. 31912274 10.1007/s10461-020-02777-6 PMC7223907

[34] Sevelius JM , Patouhas E , Keatley JG , Johnson MO . Barriers and facilitators to engagement and retention in care among transgender women living with human immunodeficiency virus. Ann Behav Med. 2014;47(1):5–16. 24317955 10.1007/s12160-013-9565-8 PMC3925767

[35] Sevelius JM , Keatley J , Calma N , Arnold E . ‘I am not a man’: trans‐specific barriers and facilitators to PrEP acceptability among transgender women. Glob Public Health. 2016;11(7–8):1060–75. 26963756 10.1080/17441692.2016.1154085 PMC10204128

[36] Veronese V , Clouse E , Wirtz AL , Thu KH , Naing S , Baral SD , et al. “We are not gays… don't tell me those things”: engaging ‘hidden’ men who have sex with men and transgender women in HIV prevention in Myanmar. BMC Public Health. 2019;19(1):1–2. 30642303 10.1186/s12889-018-6351-3 PMC6332568

[37] Sevelius JM , Keatley J , Calma N , Arnold E . ‘I am not a man’: trans‐specific barriers and facilitators to PrEP acceptability among transgender women. Glob Public Health. 2016;11(7–8):1060–75. 26963756 10.1080/17441692.2016.1154085 PMC10204128

[38] Austin A , Goodman R. The impact of social connectedness and internalized transphobic stigma on self‐esteem among transgender and gender non‐conforming adults. J Homosex. 2017;64(6):825–41. 27633046 10.1080/00918369.2016.1236587

[39] Bockting WO , Miner MH , Swinburne Romine RE , Dolezal C , Robinson BB , Rosser BS , et al. The Transgender Identity Survey: a measure of internalized transphobia. LGBT Health. 2020;7(1):15–27. 31880493 10.1089/lgbt.2018.0265 PMC6983734

[40] Rendina HJ , Cain DN , López‐Matos J , Ray M , Gurung S , Parsons JT. Measuring experiences of minority stress for transgender women: adaptation and evaluation of internalized and anticipated transgender stigma scales. Transgend Health. 2020;5(1):42–9. 32322687 10.1089/trgh.2019.0059 PMC7173692

[41] Reisner SL , Moore CS , Asquith A , Pardee DJ , Mayer KH. Gender non‐affirmation from cisgender male partners: development and validation of a brief stigma scale for HIV research with transgender men who have sex with men (trans MSM). AIDS Behav. 2020;24(1):331–43. 31865515 10.1007/s10461-019-02749-5

[42] Goldenberg T , Kahle EM , Stephenson R. Stigma, resilience, and health care use among transgender and other gender diverse youth in the United States. Transgend Health. 2020;5(3):173–81. 32923667 10.1089/trgh.2019.0074 PMC7480729

[43] Watson RJ , Veale JF , Gordon AR , Clark BA , Saewyc EM . Risk and protective factors for transgender youths' substance use. Prev Med Rep. 2019;15:100905. 31193847 10.1016/j.pmedr.2019.100905 PMC6542768

